# O001. The Italian Consensus Conference “Chronic Pain in Neurorehabilitation” - Group 29: headache and facial pain

**DOI:** 10.1186/1129-2377-16-S1-A132

**Published:** 2015-09-28

**Authors:** M Gabriella Buzzi, Vittorio Schweiger, Mariangela Berlangieri, Marco Tramontano, Mariagrazia D'Ippolito, Sara Bonazza, Rosanna Cerbo, Valerio Palmerini, Riccardo Rosa, Giorgio Sandrini, Cristina Tassorelli

**Affiliations:** Headache Centre and Post-coma Unit, IRCCS Fondazione Santa Lucia, Rome, Italy; Anesthesiology, Intensive Care and Pain Therapy Centre, Dept of Surgical Sciences, Policlinico G.B. Rossi, University of Verona, Verona, Italy; Neurorehabilitation Unit, C. Mondino National Neurological Institute, Dept of Brain and Behavioral Sciences, University of Pavia, Pavia, Italy; Pain Therapy Hub, Policlinico Umberto I, Rome, Italy

Headaches and other cranio-oro-facial pains are widely distributed in the general population. Unfortunately, there is very little evidence regarding the impact of these conditions in patients admitted to rehabilitation units, regardless of the disease or syndrome requiring rehabilitation. The availability of diagnostic and therapeutic guidelines, as well as the increasing number of data coming from controlled clinical trials, should be implemented in these patients to reduce the burden of pain and improve their global outcome.

The Italian Society for Neurorehabiltation, in collaboration with the Italian Society of Physical Medicine and Rehabilitation, has promoted the Consensus Conference on Pain with the aim to foster attention on pain also in the rehabilitative field (http://www.doloreinneuroriabilitazione.it/).

The working group has proposed the following recommendations:

- Standard methods or criteria exist to evaluate head and cranio-facial pain in terms of intensity (B);

- Standard methods exist to evaluate migraine in terms of disability (A);

- It is important to evaluate the impact of cephalic and cranio-facial pain in neurorehabilitation (D);

- Standard methods or criteria exist to diagnose head and cranio-facial pain (GL);

- It is important to identify predictive factors associated with the development of cephalic and cranio-facial pain in association with a condition requiring neurorehabilitation (D);

- Effective pharmacological treatment exists for primary headaches and for trigeminal neuralgia (GL);

- Manual therapy is indicated in the management of migraine and tension-type headache (GL);

- Manual therapy may be effective in TMD-associated pain (D);

- Botulinum toxin A is effective in the treatment of idiopathic trigeminal neuralgia (B);

- Botulinum toxin A is effective in the treatment of hemifacial spasm (B);

- Topical capsaicin is effective in chronic neuropathic pain (B);

- Evidence is needed to evaluate the impact of treating cephalic and cranio-facial pain on the outcome of patients undergoing neurorehabilitation (D).

The recommendations are presently under evaluation by the Consensus Conference panel.

